# A new tyrannosaurid (Dinosauria: Theropoda) from the Upper Cretaceous Menefee Formation of New Mexico

**DOI:** 10.7717/peerj.5749

**Published:** 2018-10-09

**Authors:** Andrew T. McDonald, Douglas G. Wolfe, Alton C. Dooley

**Affiliations:** 1 Western Science Center, Hemet, CA, USA; 2 Zuni Dinosaur Institute for Geosciences, Springerville, AZ, USA

**Keywords:** *Dynamoterror dynastes*, Tyrannosauridae, Allison member, Menefee formation

## Abstract

The giant tyrannosaurids were the apex predators of western North America and Asia during the close of the Cretaceous Period. Although many tyrannosaurid species are known from numerous skeletons representing multiple growth stages, the early evolution of Tyrannosauridae remains poorly known, with the well-known species temporally restricted to the middle Campanian-latest Maastrichtian (∼77–66 Ma). The recent discovery of a new tyrannosaurid, *Lythronax argestes*, from the Wahweap Formation of Utah provided new data on early Campanian (∼80 Ma) tyrannosaurids. Nevertheless, the early evolution of Tyrannosauridae is still largely unsampled. We report a new tyrannosaurid represented by an associated skeleton from the lower Campanian Allison Member of the Menefee Formation of New Mexico. Despite fragmentation of much of the axial and appendicular skeleton prior to discovery, the frontals, a metacarpal, and two pedal phalanges are well-preserved. The frontals exhibit an unambiguous autapomorphy and a second potential autapomorphy that distinguish this specimen from all other tyrannosaurids. Therefore, the specimen is made the holotype of the new genus and species *Dynamoterror dynastes*. A phylogenetic analysis places *Dynamoterror dynastes* in the tyrannosaurid subclade Tyrannosaurinae. Laser-scanning the frontals and creation of a composite 3-D digital model allows the frontal region of the skull roof of *Dynamoterror* to be reconstructed.

## Introduction

During most of the Late Cretaceous Epoch, the interior of North America was inundated by a shallow epicontinental seaway, with two landmasses on either side remaining as dry land: Appalachia in the east and Laramidia in the west (Fig. 1 in Sampson et al. (2010)). Tyrannosauroid theropods were the largest dinosaurian predators in Appalachia, Laramidia, and Asia during the Campanian and Maastrichtian ages (Loewen et al., 2013; Brusatte & Carr, 2016), approximately the final 15 million years of the Late Cretaceous before the K–Pg mass extinction (Cohen et al., 2013; Renne et al., 2013). The Appalachian record is sparse, consisting of two large-bodied tyrannnosauroid taxa, *Appalachiosaurus montgomeriensis* and *Dryptosaurus aquilunguis*, each known from a single associated skeleton (Carr, Williamson & Schwimmer, 2005; Brusatte, Benson & Norell, 2011).

In contrast, the Campanian–Maastrichtian record of tyrannosauroids from Asia and especially Laramidia is extensive, consisting of numerous representatives of Tyrannosauridae, the largest and most derived tyrannosauroids. The Asian record of Tyrannosauridae includes the unusual, small-bodied, longirostrine alioramins (*Alioramus remotus* (Kurzanov, 1976), *Alioramus altai* (Brusatte et al., 2009), and *Qianzhousaurus sinensis* (Lü et al., 2014)), numerous skeletons of the immense *Tarbosaurus bataar* (Hurum & Sabath, 2003; Tsuihiji et al., 2011), and the recently named large-bodied tyrannosaurid *Zhuchengtyrannus magnus* (Hone et al., 2011).

The more bountiful record of Tyrannosauridae from Laramidia includes the poorly known, small-bodied *Nanuqsaurus hoglundi* from Alaska (Fiorillo & Tykoski, 2014), and a variety of large-bodied tyrannosaurids known from multiple specimens, such as *Albertosaurus sarcophagus* (Currie, 2003; Carr, 2010), *Gorgosaurus libratus* (Currie, 2003), and *Daspletosaurus torosus* (Russell, 1970; Currie, 2003) from Alberta; *Daspletosaurus horneri* from Montana (Carr et al., 2017); *Teratophoneus curriei* from Utah (Carr et al., 2011; Loewen et al., 2013); *Bistahieversor sealeyi* from New Mexico (Carr & Williamson, 2010), which is the sister taxon of Tyrannosauridae (Carr & Williamson, 2010; Brusatte & Carr, 2016); and the latest and most colossal of the tyrannosaurids, *Tyrannosaurus rex*, known from many locations throughout the western United States and Canada (Brochu, 2003; Currie, 2003; Carr & Williamson, 2004; Sampson & Loewen, 2005). There is also the incomplete skeleton of *Labocania anomala* from Baja California (Molnar, 1974), which is of uncertain phylogenetic affinities but might be a tyrannosauroid (Holtz, 2004).

Although exceptionally rich, the tyrannosaurid record from Laramidia is temporally restricted to between approximately 77.0 Ma (*Daspletosaurus torosus* (Currie, 2003; Carr et al., 2017; Fowler, 2017)) and 66.0 Ma (*Tyrannosaurus rex* (Carr & Williamson, 2004; Holtz, 2004)), obscuring the earlier evolution of the group. The recent discovery of a new tyrannosaurid, *Lythronax argestes*, from the Wahweap Formation of Utah (Loewen et al., 2013), provided the first extensive morphological data for an early Campanian (∼80 Ma) tyrannosaurid from Laramidia. Herein we describe a new genus and species of tyrannosaurid from the lower Campanian Allison Member of the Menefee Formation in the San Juan Basin of northwestern New Mexico. The new taxon is known from an incomplete but associated skeleton including cranial and postcranial elements, and provides further insight into the morphology and diversity of tyrannosaurids from the early Campanian of Laramidia.

## Materials and Methods

The specimen described herein was collected under permit NM12-03S issued by the US Bureau of Land Management (BLM).

The electronic version of this article in portable document format will represent a published work according to the International Commission on Zoological Nomenclature (ICZN), and hence the new names contained in the electronic version are effectively published under that Code from the electronic edition alone. This published work and the nomenclatural acts it contains have been registered in ZooBank, the online registration system for the ICZN. The ZooBank LSIDs (Life Science Identifiers) can be resolved and the associated information viewed through any standard web browser by appending the LSID to the prefix http://zoobank.org/. The LSID for this publication is urn:lsid:zoobank.org:pub:E6547114-4285-49BB-AF82-1280F631748F. The online version of this work is archived and available from the following digital repositories: PeerJ, PubMed Central and CLOCKSS.

### Phylogenetic analysis

The data matrix of Carr et al. (2017) was used to test the relationships of the Allison Member tyrannosaurid; the addition of the new taxon was the only change to the matrix. The data matrix consisted of 34 taxa and 386 characters. The new taxon could be coded for only nine of the characters (S1 Codings and Measurements of UMNH VP 28348).

The matrix was analyzed in TNT 1.5 (Goloboff & Catalano, 2016). All 49 characters treated as ordered by Carr et al. (2017) were again treated as ordered. We followed the procedure of Brusatte & Carr (2016). *Allosaurus* was designated as the outgroup. The matrix was first analyzed using a New Technology Search, with the default parameters for sectorial search, ratchet, tree drift, and tree fusion; a random seed of 1; 10 replicates; and the number of times to find a minimum length tree set at 10. This search examined 69,709,132 rearrangements and recovered 12 most parsimonious trees of 813 steps, consistency index of 0.552, and retention index of 0.803. These 12 trees were then examined using the tree bisection reconnection swapping algorithm, which examined 279,310 rearrangements and recovered 18 most parsimonious trees. The strict consensus of these 18 trees was then derived in TNT. Bremer support values for the strict consensus cladogram were then calculated in TNT.

## Results

### Systematic paleontology

Dinosauria Owen, 1842, *sensu* Baron, Norman & Barrett, 2017
Theropoda Marsh, 1881, *sensu* Baron, Norman & Barrett, 2017
Coelurosauria Huene, 1914, *sensu* Sereno, McAllister & Brusatte, 2005
Tyrannosauroidea Osborn, 1906, *sensu* Walker, 1964; Sereno, McAllister & Brusatte, 2005
Tyrannosauridae Osborn, 1906, *sensu* Sereno, McAllister & Brusatte, 2005
Tyrannosaurinae Osborn, 1906, *sensu* Matthew & Brown, 1922; Sereno, McAllister & Brusatte, 2005

*Dynamoterror dynastes* gen. et sp. nov.

Holotype: UMNH VP 28348, incomplete associated skeleton including the left and right frontals, four fragmentary vertebral centra, fragments of dorsal ribs, right metacarpal II, supraacetabular crest of the right ilium, unidentifiable fragments of long bones, phalanx 2 of left pedal digit IV, and phalanx 4 of left pedal digit IV.

Etymology: *Dynamoterror* is derived from the transliterated Greek word *dynamis* (“power”) and the Latin word *terror*. The specific name, *dynastes*, is a Latin word meaning “ruler.” The intended meaning of the binomen is “powerful terror ruler.” The name also honors the binomen “*Dynamosaurus imperiosus*” (Osborn, 1905), a junior synonym of *Tyrannosaurus rex* (Osborn, 1905, 1906), but a particular childhood favorite of the lead author.

Locality: UMNH VP 28348 was collected in San Juan County, New Mexico, on land administered by the US BLM. Precise locality data are on file at UMNH and the BLM.

Horizon: UMNH VP 28348 was collected from outcrops of the Juans Lake Beds (Miller, Carey & Thompson-Rizer, 1991), upper part of the Allison Member, Menefee Formation; lower Campanian, Upper Cretaceous. Lucas et al. (2005) produced a radioisotopic date of 78.22 ± 0.26 Ma from a bentonite layer near the top of the Menefee Formation in the Gallina hogback in the eastern part of the San Juan Basin. In the part of the San Juan Basin where UMNH VP 28348 was collected, the overlying Cliff House Sandstone contains fossils of the ammonoid *Baculites perplexus* (Siemers & King, 1974), corresponding to between 78.0 and 78.5 Ma (Molenaar et al., 2002). According to the regional stratigraphic correlation chart of Molenaar et al. (2002), the Menefee Formation spans approximately 84.0–78.5 Ma, based upon correlations with marine biostratigraphic zones. This age range corresponds to uppermost Santonian—middle Campanian (Cohen et al., 2013).

Specific diagnosis (as for genus by monotypy): tyrannosaurine tyrannosaurid distinguished by two autapomorphies on the frontals: (1) prefrontonasal and prefrontolacrimal processes are in close proximity, separated only by a shallow notch; and (2) subrectangular, concave, laterally projecting caudal part of the postorbital suture, separated from the rostral part by a deep groove. The second autapomorphy should be treated as provisional, given the ontogenetic variation observed in this region of the frontal in other tyrannosaurids (Carr & Williamson, 2004) (see description of the lateral surface of the frontal below). In the context of the phylogenetic analysis of Carr et al. (2017), which is used herein, UMNH VP 28348 exhibits a feature that supports its affinities among derived tyrannosauroids (156^1^, “frontal, dorsotemporal fossa, medial extension, dorsal view: meets opposing fossa at the midline”; also present in *Timurlengia euotica*, *Xiongguanlong baimoensis*, *B. sealeyi*, and Tyrannosauridae), and a feature identified by Carr et al. (2017) as an unambiguous synapomorphy of “derived tyrannosaurines” (157^1^, “frontal, sagittal crest, form, dorsal and lateral views: present and pronounced (dorsoventrally tall), single structure”).

### Description

UMNH VP 28348 was collected at a single locality; as there is no duplication, morphological inconsistency, or size incompatibility among the identifiable elements, all bones collected from the site are regarded as pertaining to a single tyrannosaurid individual. All elements were collected as float in 2012. A test excavation that same year and subsequent visits in 2013 and 2018 did not reveal additional bones at the site. Measurements of select cranial and appendicular elements of UMNH VP 28348 are available in the supplementary information (S1 Codings and Measurements of UMNH VP 28348). Only those elements of UMNH VP 28348 that are identifiable based upon comparison with other tyrannosaurids are described.

Previous discoveries of tyrannosaurids in the Menefee Formation include tooth fragments and a metatarsal reported by Hunt & Lucas (1993), and another tooth fragment reported by Lewis (2006). Lack of overlapping material precludes referral of any of these elements to *Dynamoterror dynastes*. UMNH VP 28348 is the first associated tyrannosaurid skeleton reported from the Menefee Formation.

#### Frontals

**Figure 1:**
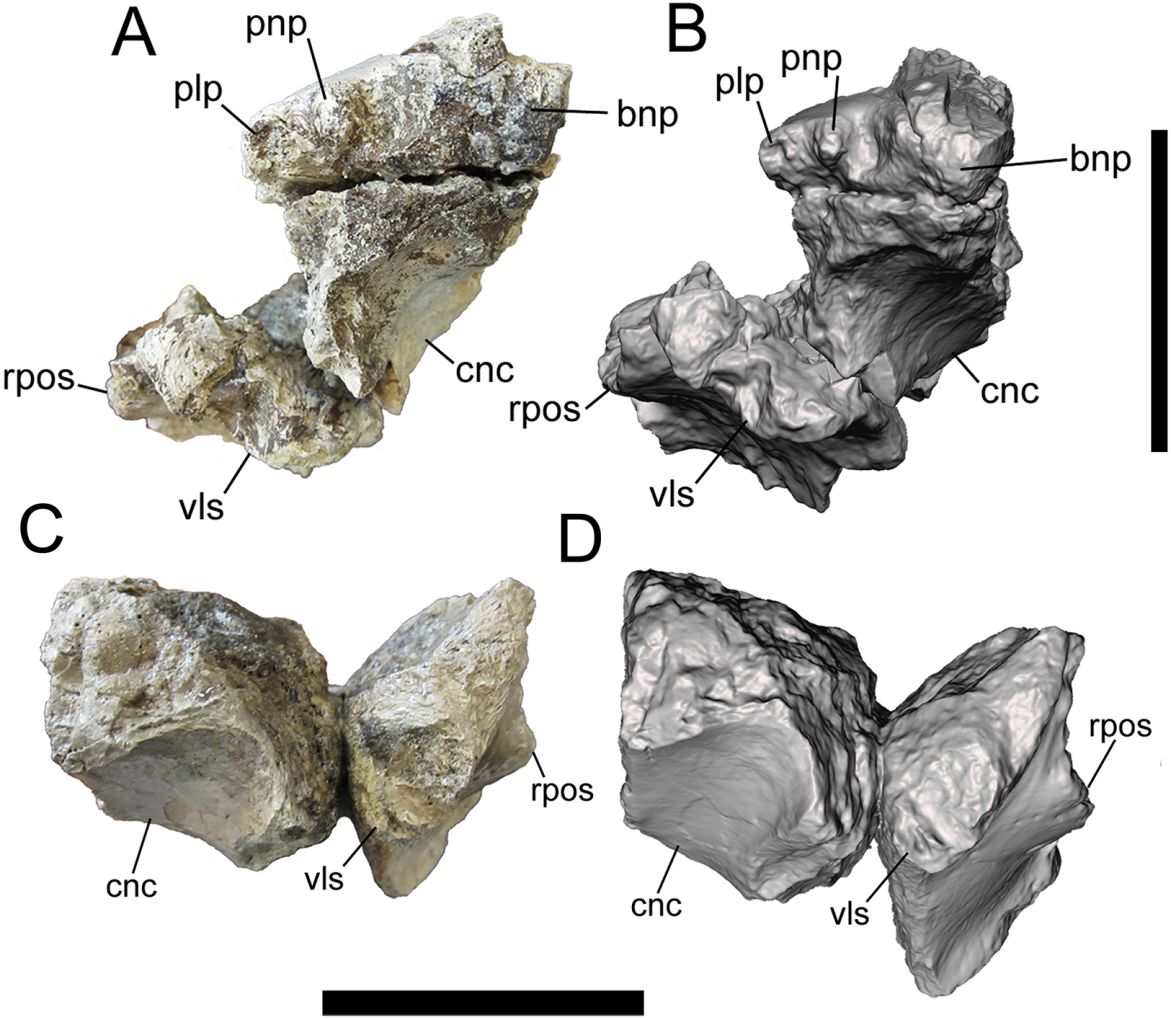
Frontals of UMNH VP 28348 in rostral view. Photographs and 3-D models of right (A, B) and left (C, D) frontals of UMNH VP 28348. Abbreviations: bnp, basal of nasal process; cnc, caudal extent of nasal cavity; plp, prefrontolacrimal process; pnp, prefrontonasal process; rpos, rostral part of postorbital suture; vls, ventrolateral part of lacrimal suture. Scale bars equal five cm.

**Figure 2:**
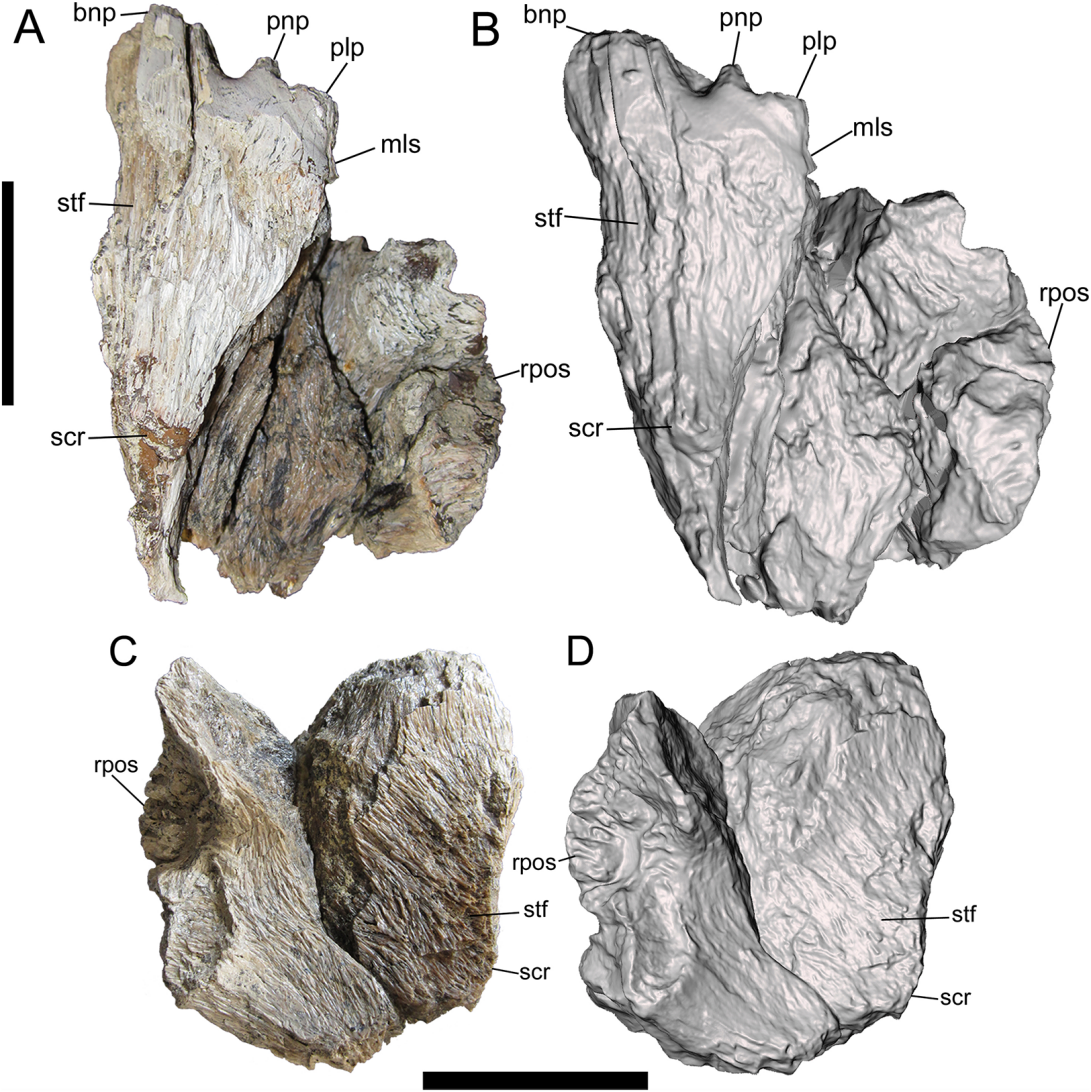
Frontals of UMNH VP 28348 in dorsal view. Photographs and 3-D models of right (A, B) and left (C, D) frontals of UMNH VP 28348. Abbreviations: bnp, basal of nasal process; mls, medial-most point of lacrimal suture; plp, prefrontolacrimal process; pnp, prefrontonasal process; rpos, rostral part of postorbital suture; scr, sagittal crest; stf, supratemporal fossa. Scale bars equal five cm.

**Figure 3:**
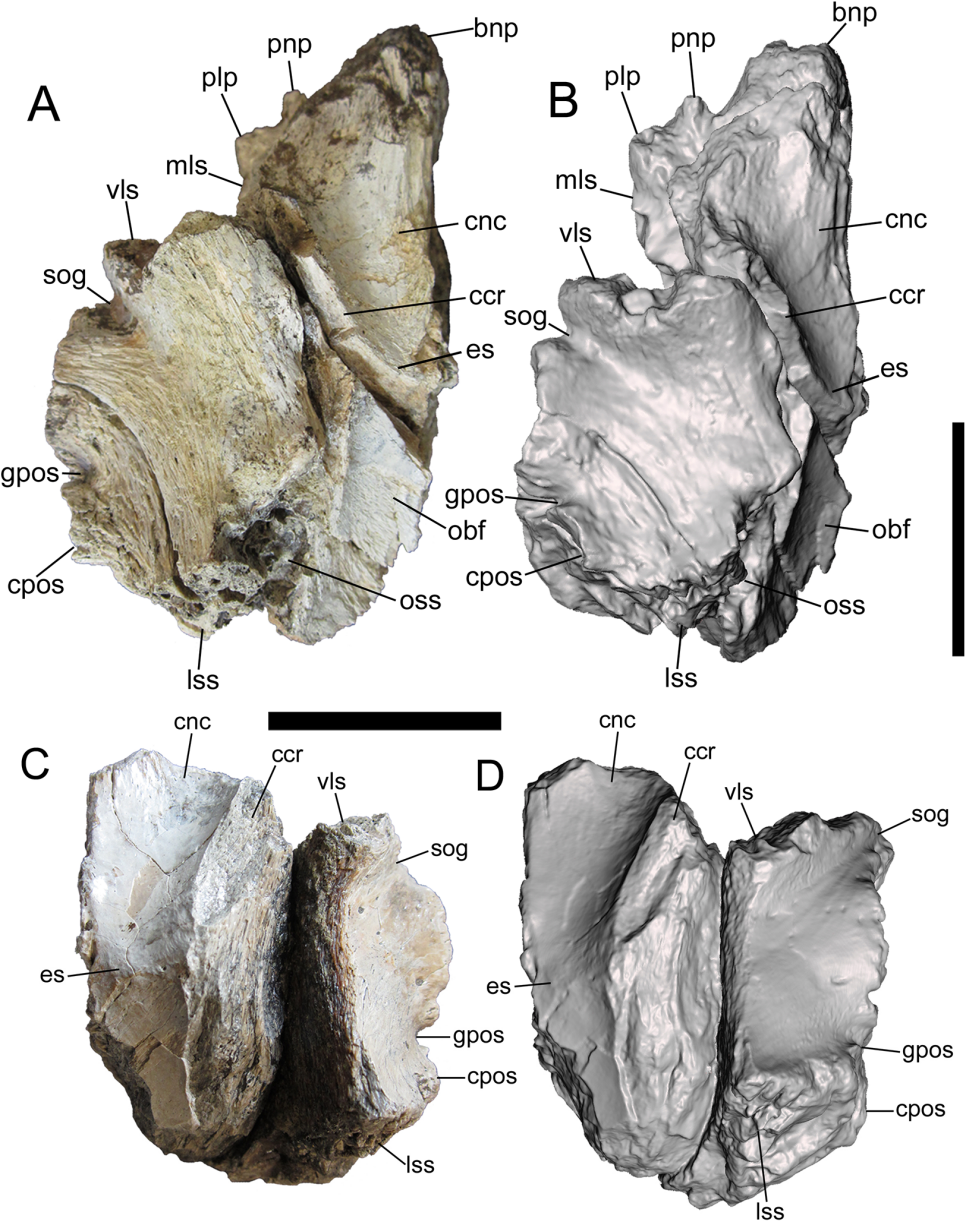
Frontals of UMNH VP 28348 in ventral view. Photographs and 3-D models of right (A, B) and left (C, D) frontals of UMNH VP 28348. Abbreviations: bnp, basal of nasal process; ccr, crista cranii; cnc, caudal extent of nasal cavity; cpos, caudal part of postorbital suture; es, ethmoid scar; gpos, groove between rostral and caudal parts of postorbital suture; lss, laterosphenoid suture; mls, medial-most point of lacrimal suture; obf, olfactory bulb fossa; oss, orbitosphenoid suture; plp, prefrontolacrimal process; pnp, prefrontonasal process; sog, supraorbital groove; vls, ventrolateral part of lacrimal suture. Scale bars equal five cm.

**Figure 4:**
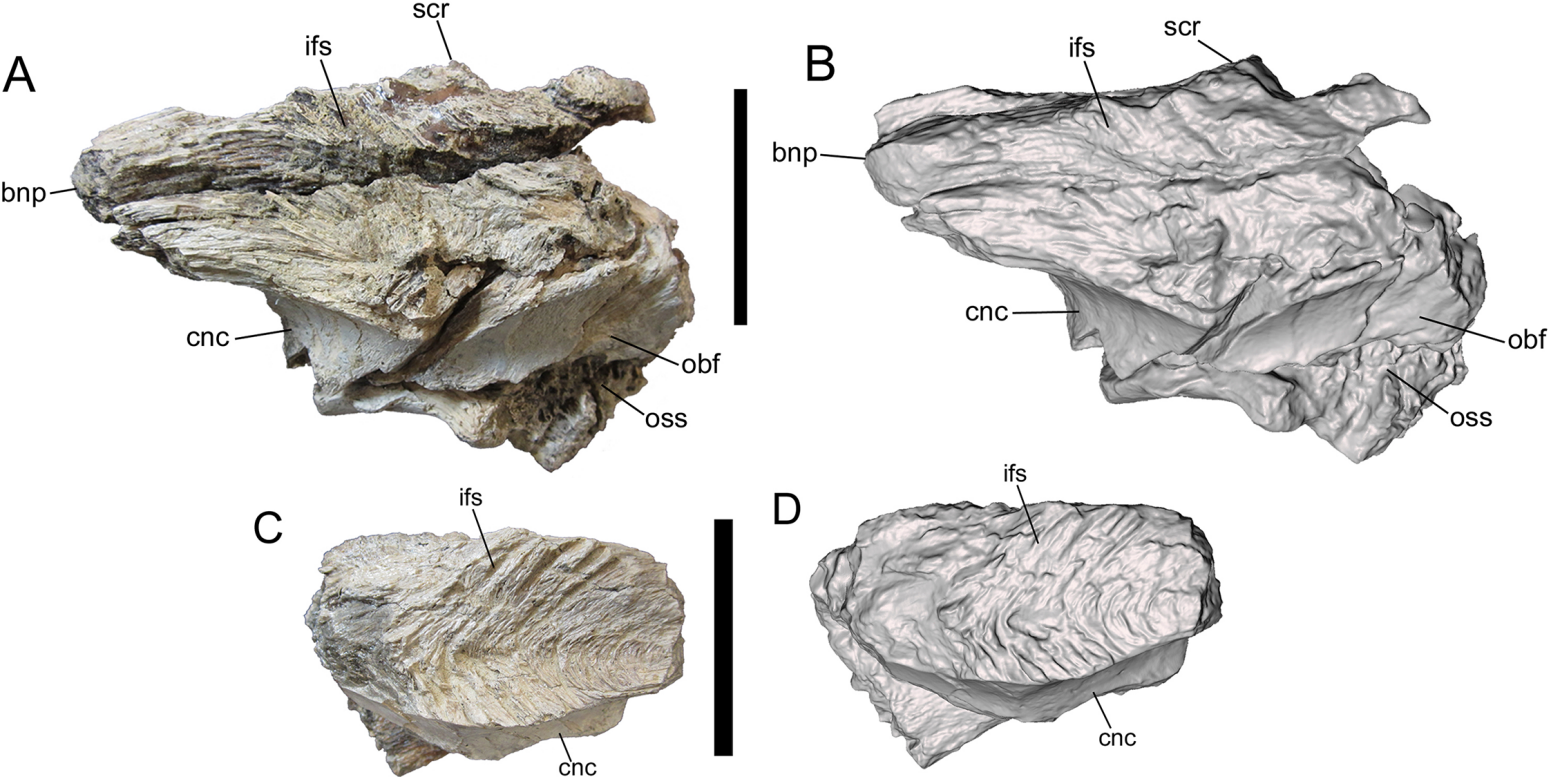
Frontals of UMNH VP 28348 in medial view. Photographs and 3-D models of right (A, B) and left (C, D) frontals of UMNH VP 28348. Abbreviations: bnp, basal of nasal process; cnc, caudal extent of nasal cavity; ifs, interfrontal suture; obf, olfactory bulb fossa; oss, orbitosphenoid suture; scr, sagittal crest. Scale bars equal five cm.

**Figure 5:**
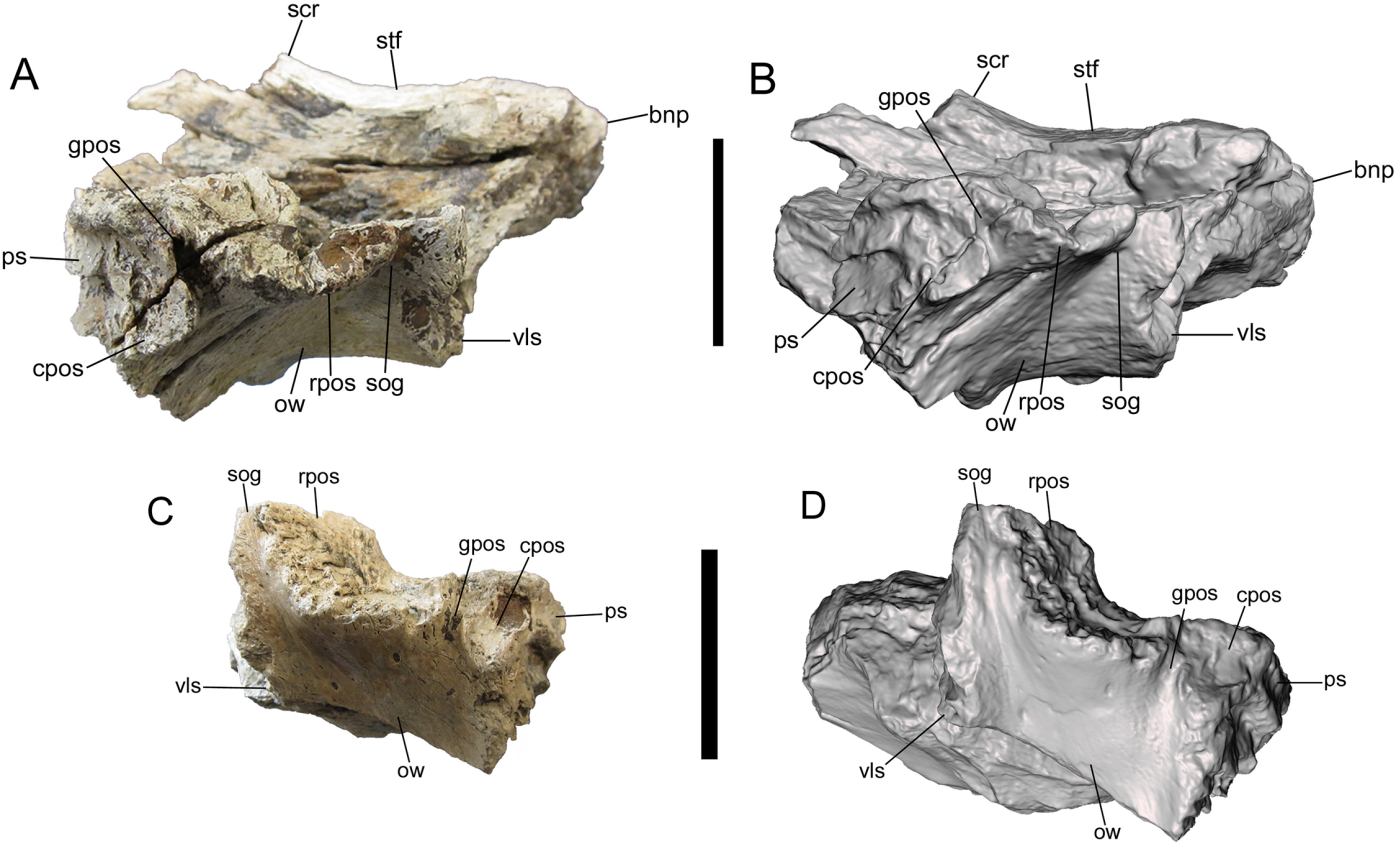
Frontals of UMNH VP 28348 in lateral view. Photographs and 3-D models of right (A, B) and left (C, D) frontals of UMNH VP 28348. Abbreviations: bnp, basal of nasal process; cpos, caudal part of postorbital suture; gpos, groove between rostral and caudal parts of postorbital suture; ow, orbital wall; ps, parietal suture; rpos, rostral part of postorbital suture; scr, sagittal crest; sog, supraorbital groove; stf, supratemporal fossa; vls, ventrolateral part of lacrimal suture. Scale bars equal five cm.

**Figure 6:**
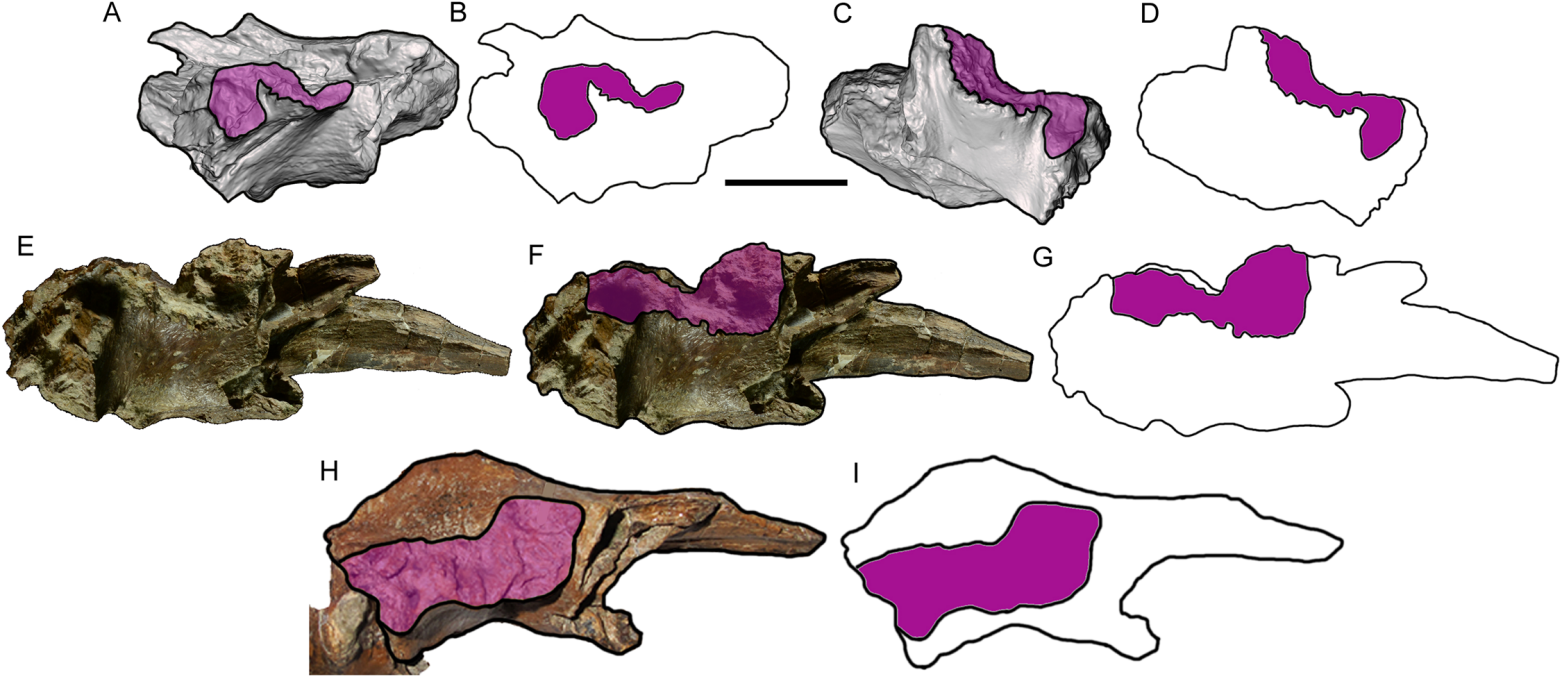
Comparison among derived tyrannosaurine frontals. Digital models and line drawings of the right (A, B) and left (C, D) frontals of *Dynamoterror dynastes*, UMNH VP 28348, in lateral view. The postorbital suture is highlighted in purple. Scale bar equals five cm for A–D. BYU 8120/9396, right frontal of *Teratophoneus curriei* in lateral view, shown as a photograph (E), photograph with the postorbital suture highlighted in purple (F), and a line drawing with the postorbital suture highlighted in purple (G). Photograph of BYU 8120/9396 courtesy of Rod Scheetz (BYU) and is used here with permission. Additional anatomical information from UMNH VP 16690 (Fig. 3E in Loewen et al. (2013)). UMNH VP 20200, right frontal of *Lythronax argestes* in lateral view, shown as an image with the postorbital suture highlighted in purple (H) (modified from Fig. 2E in Loewen et al. (2013)), and a line drawing with the postorbital suture highlighted in purple (I).

**Figure 7:**
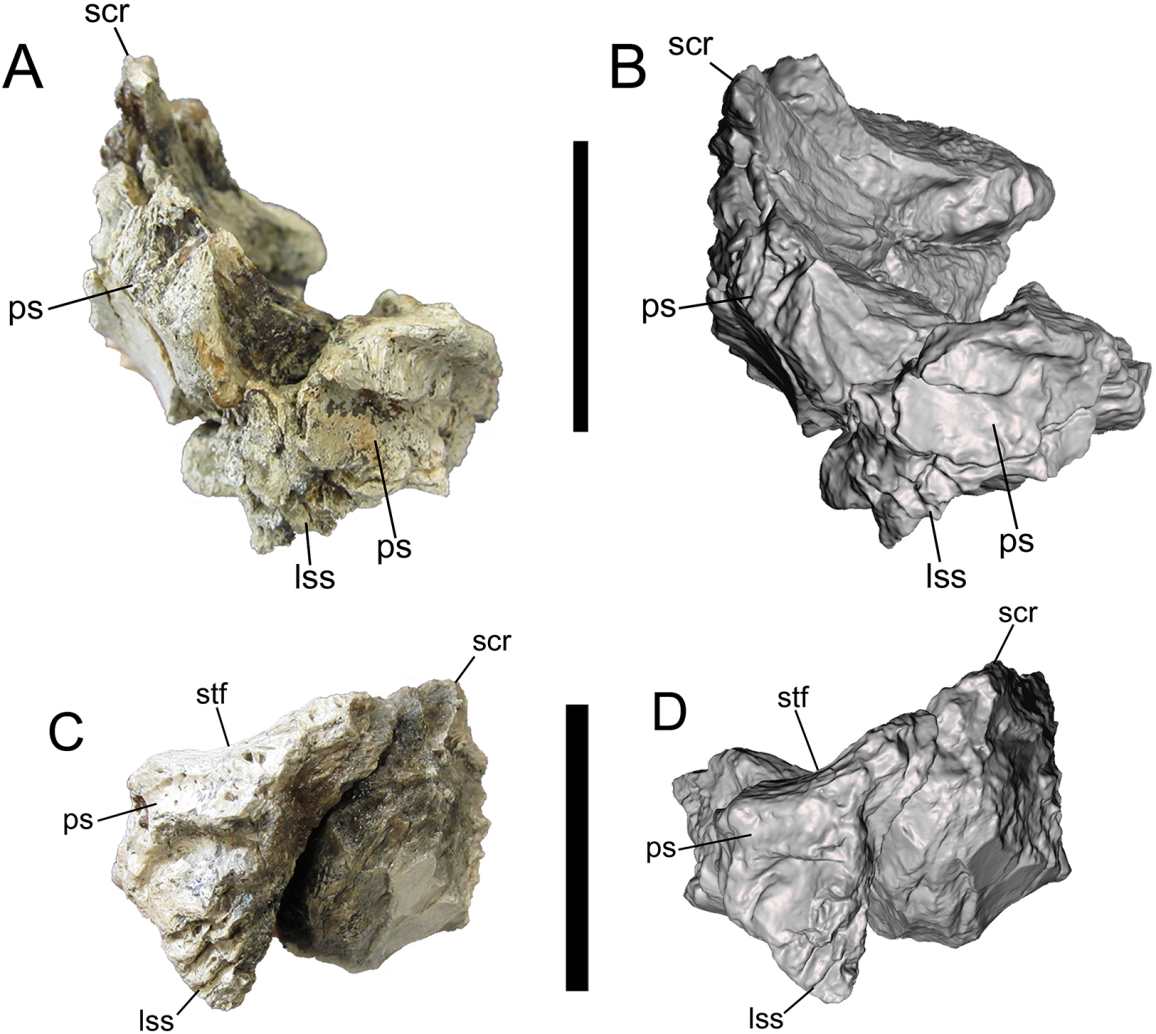
Frontals of UMNH VP 28348 in caudal view. Photographs and 3-D models of right (A, B) and left (C, D) frontals of UMNH VP 28348. Abbreviations: lss, laterosphenoid suture; ps, parietal suture; scr, sagittal crest; stf, supratemporal fossa. Scale bars equal five cm.

The right and left frontals both are incomplete; however, between them, they exhibit nearly the entire frontal morphology, with the exception of the rostral end of the nasal process (Figs. 1–7). Many fine details are preserved, allowing thorough comparisons with other tyrannosaurid frontals. Unfortunately, no frontals are known for the Late Cretaceous Appalachian outgroups of Tyrannosauridae, *Appalachiosaurus montgomeriensis* and *Dryptosaurus aquilunguis* (Carr, Williamson & Schwimmer, 2005; Brusatte, Benson & Norell, 2011). However, comparisons were possible with *B. sealeyi* (Lehman & Carpenter, 1990; Carr & Williamson, 2000, 2010), which is the closest outgroup of Tyrannosauridae (Carr & Williamson, 2010; Brusatte & Carr, 2016), and with numerous members of Tyrannosauridae, including the albertosaurines *Albertosaurus sarcophagus* and *G. libratus* (Currie, 2003) and the tyrannosaurines *Alioramus altai* (Bever et al., 2013), *Daspletosaurus* sp. (Currie, 2003), *L. argestes* (UMNH VP 20200) (Loewen et al., 2013), *Teratophoneus curriei* (BYU 8120/9396, UMNH VP 16690) (Carr et al., 2011; Loewen et al., 2013), *N. hoglundi* (Fiorillo & Tykoski, 2014), *Tarbosaurus bataar* (Hurum & Sabath, 2003), *Tyrannosaurus rex* (LACM 23845, LACM 150167) (Osborn, 1912; Brochu, 2003; Carr & Williamson, 2004; Larson, 2008), and an unnamed tyrannosaurine from the Aguja Formation of Texas (Lehman & Wick, 2012).

The length, width, and depth of the frontals of UMNH VP 28348 were measured after the methodology of Currie (2003). The overall dimensions of the right frontal of UMNH VP 28348 are similar to those of LACM 23845 (Table 2 in Currie (2003)), a subadult specimen of *Tyrannosaurus rex* (Molnar, 1980; Carr & Williamson, 2004) (S1 Codings and Measurements of UMNH VP 28348). Although complete length cannot be measured for the frontals of UMNH VP 28348 due to breakage of the nasal processes, width and depth can be measured for the right frontal. The ratio of depth to width for the right frontal is 0.57, comparable to frontals of subadult and adult *Daspletosaurus* sp. (0.50–0.66) and *Tyrannosaurus rex* (0.59–0.67, including LACM 23845 (0.62)), calculated from the measurements provided by Currie (2003; Table 2). In tyrannosaurids, the depth of the frontal exhibits positive allometry relative to width during the course of ontogeny (Currie, 2003). Coupled with the measurements and depth/width ratio, this suggests that UMNH VP 28348 represents a subadult or adult individual.

The frontals are described together as a single unit, though it is noted whether a feature is most clearly preserved on the right, left, or both frontals. Given the complexity of the morphology of the frontals, the description is divided into six sections, detailing the rostral, dorsal, ventral, medial, lateral, and caudal surfaces.

##### Rostral surface

**Figure 8:**
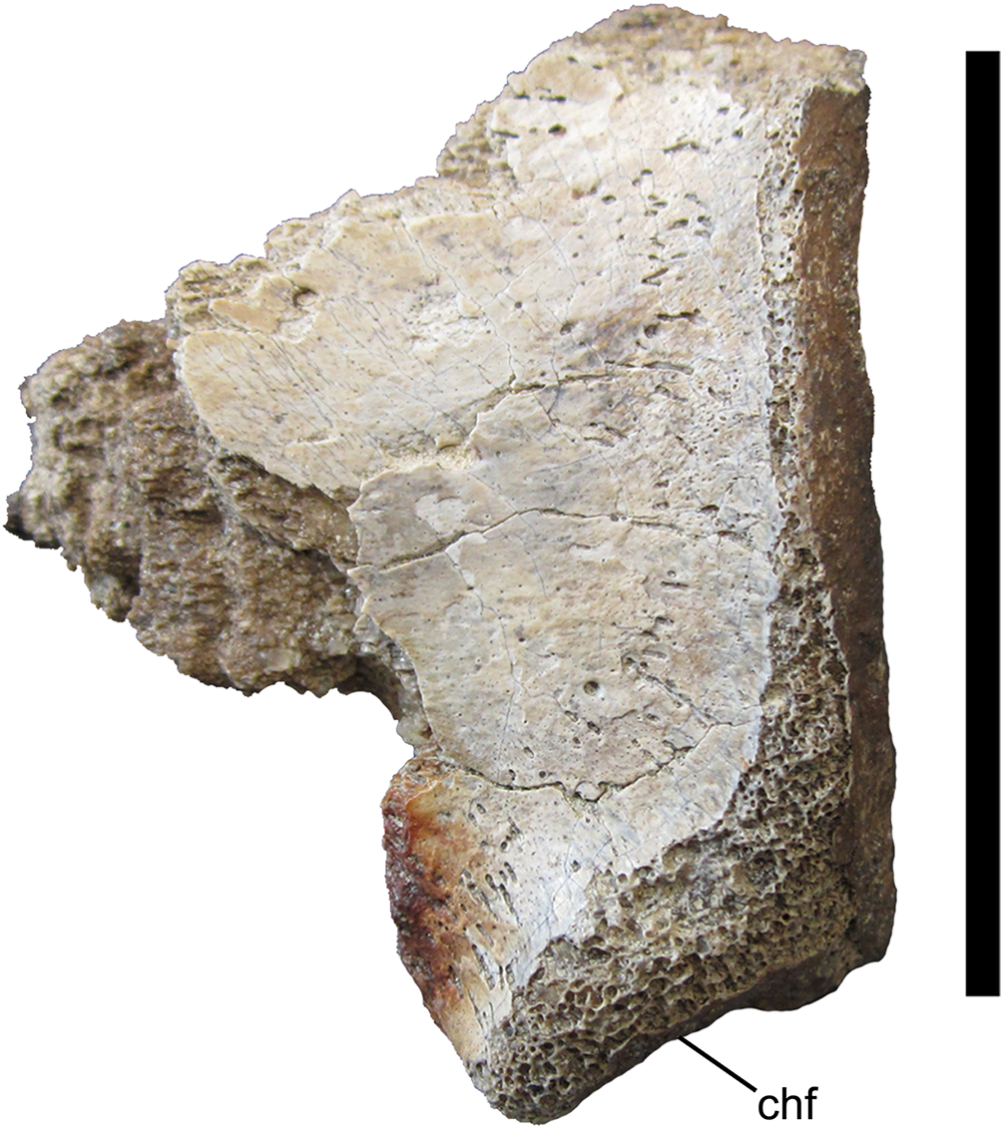
Middle caudal centrum of UMNH VP 28348. Incomplete middle caudal centrum in left lateral view. Abbreviations: chf, chevron facet. Scale bar equals five cm.

The medial-most feature on the rostral surface is the base of the nasal process. The nasal process projects rostrally, but is missing its rostral end (right frontal) (Figs. 1A, 1B, 2A and 2B). Lateral to the base of the nasal process is a deep notch that marks the caudal-most point of the contact with the nasal (right frontal). Lateral to this deep notch is the small, conical, rostrally projecting prefrontonasal process (right frontal) (Figs. 1A, 1B, 2A and 2B), as in *Teratophoneus curriei* (BYU 8120/9396) and *L. argestes* (UMNH VP 20200) (Fig. 2E in Loewen et al. (2013)). Lateral to the prefrontonasal process is another, shallower notch, which marks the location of prefrontal exposure on the skull roof. Lateral to this notch is a circular broken surface that represents the base of another small conical process similar to the prefrontonasal process (right frontal) (Figs. 1A, 1B, 2A and 2B). This more laterally situated small conical process is the prefrontolacrimal process. This small prefrontolacrimal process differs from the much larger, rostrocaudally long prefrontolacrimal processes that separate the prefrontal and lacrimal sutures in *Teratophoneus curriei* (BYU 8120/9396) (Fig. 3E in Loewen et al. (2013)) and *N. hoglundi* (Figs. 3F–3I in Fiorillo & Tykoski (2014)). It is similar to the rostrocaudally short prefrontolacrimal processes of *L. argestes* (UMNH VP 20200) (Fig. 2E in Loewen et al. (2013)), *Daspletosaurus* sp. (Figs. 20A, 20C in Currie (2003)), *Tyrannosaurus rex* (LACM 23845, LACM 150167) (Larson, 2008), and *Tarbosaurus bataar* (Figs. 8, 17 in Hurum & Sabath (2003)). The close proximity of the prefrontonasal and prefrontolacrimal processes, which are separated only by a narrow notch, is unique to UMNH VP 28348 and is an autapomorphy of *Dynamoterror dynastes*.

Lateral to the prefrontolacrimal process is a smooth surface that extends caudally and then begins to curve laterally, where it is truncated by a broken surface (right frontal) (Figs. 2A and 2B). This surface marks the medial-most point of the lacrimal suture. Lateral to this surface, the right frontal is missing much of the lacrimal suture. However, the ventrolateral portion of the lacrimal suture is preserved on the right frontal (Figs. 1A and 2B), and to a lesser extent on the left frontal (Figs. 1C and 1D). This part of the suture forms a dorsoventrally deep, rostrally facing cup-like structure that is rostrally concave and ventrolaterally convex, as in *L. argestes* (UMNH VP 20200) (Figs. 2E, 5 in Loewen et al. (2013)), *Teratophoneus curriei* (BYU 8120/9396) (Fig. 3E in Loewen et al. (2013)), *Daspletosaurus* sp. (Fig. 20 in Currie (2003)), *Tyrannosaurus rex* (LACM 150167) (Larson, 2008), *Tarbosaurus bataar* (Figs. 8, 17 in Hurum & Sabath (2003)), and *N. hoglundi* (Figs. 3F–I, 4 in Fiorillo & Tykoski (2014)). In the Aguja tyrannosaurine, the lacrimal contact is a deep, rostroventrally directed recess (Figs. 3, 4 in Lehman & Wick (2012)).

##### Dorsal surface

Only the rostromedial region of the dorsal surface is preserved on the right frontal (Figs. 2A and 2B). The left frontal preserves much of the dorsal surface, though weathering has stripped it of the outermost layer of cortical bone (Figs. 2C and 2D). However, as shown by preserved patches of complete bone surface near the lateral margin, only a fraction of a millimeter of thickness is missing from the dorsal surface of the left frontal.

On the dorsal surface of the right frontal, there is a slight swelling immediately caudal to the nasal, prefrontonasal, and prefrontolacrimal processes (Figs. 2A and 2B). Caudal to this swelling, the dorsal surface of the right frontal becomes gently concave and begins to slope caudolaterally to form the frontal portion of the supratemporal fossa (Figs. 2A and 2B). The rest of the frontal portion of the supratemporal fossa is preserved on the left frontal, on which the dorsal surface slopes caudolaterally toward the parietal suture where it formed a contiguous supratemporal fossa with the dorsolateral surface of the parietal (Figs. 2C and 2D).

On both frontals, the dorsal surface rises caudomedially to form the frontal portion of the sagittal crest (Fig. 2). The sagittal crest is better preserved on the right frontal. Although broken caudally, the height of the preserved portion above the supratemporal fossa and the curvature of the bone grain indicates that the crest would have continued to rise beyond the damaged surface and been very tall. A prominent sagittal crest that extends far onto the frontals is also present in the tyrannosaurines *Daspletosaurus* sp. (Fig. 20B in Currie (2003)), *Tyrannosaurus rex* (LACM 23845, LACM 150167) (Fig. 20 in Brochu (2003)) (Carr & Williamson, 2004; Larson, 2008), *Teratophoneus curriei* (BYU 8120/9396) (Fig. 3E in Loewen et al. (2013)), *L. argestes* (UMNH VP 20200) (Fig. 2E in Loewen et al. (2013)), *N. hoglundi* (Fig. 4 in Fiorillo & Tykoski (2014)), *Tarbosaurus bataar* (Fig. 17 in Hurum & Sabath (2003)), and the Aguja tyrannosaurine (Figs. 3, 4 in Lehman & Wick (2012)), as well as *B. sealeyi* (Fig. 2 in Lehman & Carpenter (1990); Fig. 1A in Carr & Williamson (2010)). This morphology differs from the albertosaurines *Albertosaurus sarcophagus* and *G. libratus*, which have much lower sagittal crests on the frontals (Currie, 2003). The morphology of the sagittal crest suggests that UMNH VP 28348 represents a tyrannosaurine.

##### Ventral surface

The detailed description of the frontals of *Alioramus altai* (see Fig. 9 in Bever et al. (2013)) was useful for interpreting the ventral surfaces of the frontals of UMNH VP 28348. Details of the rostral region of the ventral surface are well preserved on both frontals. This region is dominated by a rostrocaudally elongate, mediolaterally wide fossa that is defined rostrally by the prefrontal suture, medially by the interfrontal suture, laterally by the crista cranii, and caudally by the ethmoid scar (Fig. 3). When the frontals are placed in articulation, the right and left fossae form the caudal extent of the olfactory region of the nasal cavity, which was lined with mucous membrane when the animal was alive (Witmer & Ridgely, 2009; Bever et al., 2013). Lateral to the fossa is the crista cranii, a thick, sharply defined ridge that extends rostrolaterally to caudomedially, delineating the ventral and lateral surfaces of the frontal (Fig. 3).

The rostral fossa is separated from the olfactory bulb fossa by the ethmoid scar, a subtle mediolaterally oriented ridge that branches off of the crista cranii (Fig. 3). Only a small portion of the olfactory bulb fossa is preserved on the left frontal, while the entire fossa and the surrounding features of the caudal region of the ventral surface are intact on the right frontal. The large olfactory bulb fossa is defined rostrally by the ethmoid scar, medially by the interfrontal suture, laterally by the crista cranii and orbitosphenoid suture, and caudally by the parietal suture (Figs. 3A and 3B). The orbitosphenoid suture is a subcircular, ventromedially facing concavity lined with a complex set of delicate ridges, bumps, and depressions. The orbitosphenoid suture widens caudally where it merges with the laterosphenoid suture on the caudoventral surface of the frontal.

##### Medial surface

The medial surface is occupied entirely by the flat, vertical interfrontal suture (Fig. 4). The suture itself is better preserved on the left frontal, and consists of a series of overlapping fine, V-shaped ridges with the V’s opening rostrally.

##### Lateral surface

The details of the lateral surface are well-preserved on both frontals and include a number of characters of diagnostic and comparative value. The lacrimal and postorbital sutures are separated by a narrow, deep, vertical groove (“supraorbital groove” of Loewen et al. (2013)) (Fig. 5). Similar supraorbital grooves are observable in *L. argestes* (UMNH VP 20200) (Fig. 2E in Loewen et al. (2013)), *Teratophoneus curriei* (BYU 8120/9396) (Fig. 3E in Loewen et al. (2013)), *Tyrannosaurus rex* (LACM 150167), *N. hoglundi* (Fig. 3F–I in Fiorillo & Tykoski (2014)), *Tarbosaurus bataar* (Fig. 8A, B in Hurum & Sabath (2003)), *B. sealeyi* (Fig. 2 “cleft in frontal” in Lehman & Carpenter (1990)), and the albertosaurine *Albertosaurus sarcophagus* (Fig. 7 in Currie (2003)). The supraorbital groove grades into the orbital wall ventrally.

The postorbital suture is divided into a large rostral part and a smaller caudal part. The ventral margin of the rostral part is convex and projects farther laterally than the dorsal margin, forming a deeply concave sutural surface (Figs. 2 and 5). The rostrodorsal region of the rostral part projects dorsally, such that the rostrodorsal margin is elevated far above the caudodorsal margin and the caudodorsal margin is deeply concave (Figs. 5C and 5D). Ventral to the rostral part of the postorbital suture is the deep orbital wall, which is entirely visible in lateral view (Fig. 5).

The rostral and caudal parts of the postorbital suture are separated by a deep, smooth, caudally inclined groove that opens ventrally onto the orbital wall and is roofed dorsally by the dorsal surface of the frontal. The groove is present on both frontals but is better preserved on the right (Fig. 5). While this groove might be homologous to the open foramen that penetrates the dorsal skull roof in *Tyrannosaurus rex* (Fig. 3 in Brochu (2003)) and/or the neurovascular foramen identified in the Aguja tyrannosaurine (Figs. 3, 4 in Lehman & Wick (2012)), these possibilities must await testing by the discovery of further material.

The caudal part of the postorbital suture is a subrectangular, slightly concave structure that is delineated rostrally by a rostrolaterally projecting rim that separates it from the aforementioned deep groove, ventrally by a ventrolaterally projecting rim that separates it from the orbital wall, dorsally by a laterally projecting rim that separates it from the dorsal surface of the frontal, and caudally by a more subtle and irregular rim that also marks the lateral-most point of the parietal suture (Fig. 5). This subrectangular, concave, laterally projecting caudal part appears to be unique to UMNH VP 28348 and is proposed as an autapomorphy of *Dynamoterror dynastes*, differing from the continuous rostral and caudal parts of the postorbital suture in *B. sealeyi* (Fig. 2 in Lehman & Carpenter (1990); Fig. 4 in Lehman & Wick (2012)); the albertosaurines *Albertosaurus sarcophagus* and *G. libratus* (Currie, 2003); and the tyrannosaurines *Tyrannosaurus rex* (LACM 150167) (Fig. 7 in Osborn (1912)) (Larson, 2008), *L. argestes* (UMNH VP 20200) (Fig. 2E in Loewen et al. (2013)), *Teratophoneus curriei* (BYU 8120/9396, UMNH VP 16690) (Fig. 3E in Loewen et al. (2013)), *Daspletosaurus* sp. (Currie, 2003), *Tarbosaurus bataar* (Fig. 17C, D in Hurum & Sabath (2003)), and the Aguja tyrannosaurine (Figs. 3, 4 in Lehman & Wick (2012)). However, it should be noted that the region of the postorbital suture is subject to ontogenetic variation, particularly in changing from an area of grooves and ridges to a peg-in-socket morphology (Carr & Williamson, 2004). UMNH VP 28348 exhibits the latter morphology. This region of the frontal is damaged in the only known specimen of *N. hoglundi* (Figs. 3, 4 in Fiorillo & Tykoski (2014)). Overall, the frontals of UMNH VP 28348 are similar to those of other tyrannosaurines, such as *Teratophoneus curriei* and *L. argestes*, but differ in the aforementioned potentially autapomorphic morphology of the caudal part of the postorbital suture (Fig. 6), though this feature should be regarded with caution.

##### Caudal surface

The caudal surface is occupied by two major features, the laterosphenoid and parietal sutures, which are preserved on both frontals. The laterosphenoid suture faces caudoventrally, and is ventral to the parietal suture and caudolateral to the orbitosphenoid suture (Fig. 7). The parietal suture begins caudomedial to the caudal part of the postorbital suture and extends caudomedially before curving rostromedially and dorsally toward the base of the sagittal crest.

#### Middle caudal centrum

Of the four fragments of vertebral centra preserved in UMNH VP 28348, only one can be placed in the vertebral column with any precision. This fragment was identified as part of the centrum of a middle caudal vertebra. As on the middle caudal centra of *Tyrannosaurus rex* (Figs. 60K–Q in Brochu (2003)), that of UMNH VP 28348 exhibits a pronounced, caudoventrally directed chevron articulation facet that is offset from the ventral margin of the caudal face of the centrum (Fig. 8).

#### Right metacarpal II

**Figure 9:**
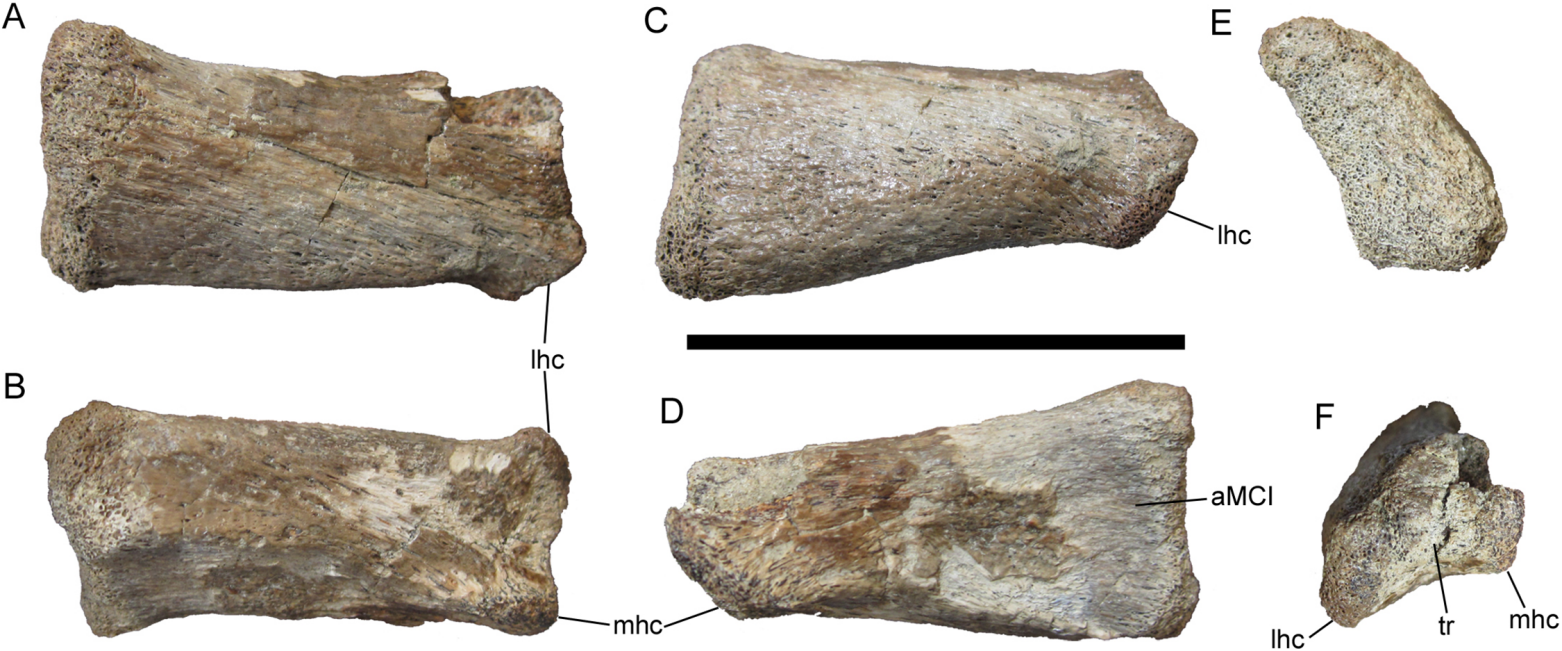
Right metacarpal II of UMNH VP 28348. Right metacarpal II in (A) dorsal; (B) ventral; (C) lateral; (D) medial; (E) proximal; and (F) distal views. Abbreviations: aMC I, surface for articulation with metacarpal I; lhc, lateral hemicondyle; mhc, medial hemicondyle; tr, trochlea. Scale bar equals five cm.

Right metacarpal II was identified based upon comparisons with the nearly complete, articulated right manus of *Tyrannosaurus rex* (Figs. 88A–D, 89 in Brochu (2003)) and left manus of *Daspletosaurus torosus* (Plates III, IV in Russell (1970)). MC II is overall nearly straight, with only a slight medial curvature toward the distal end in dorsal and ventral views (Figs. 9A and 9B). The proximal articular surface is slightly concave in lateral and medial views (Figs. 9C and 9D). In proximal view, the proximal articular surface tapers and curves medially toward its dorsal margin (Fig. 9E). The ventral surface of the shaft of MC II is flat and widens toward the distal articular surface (Fig. 9B). The dorsal and lateral surfaces of MC II are indistinct and form a gently convex surface (Figs. 9A and 9C). No articulation surface for MCIII is apparent on the lateral surface, unlike *Tyrannosaurus rex*, which exhibits a pronounced sulcus on the lateral surface of MCII near the proximal end (Brochu, 2003). The medial surface of MC II is broad and gently concave, forming a surface for articulation with the lateral surface of MC I (Fig. 9D). The distal articular surface is composed of lateral and medial hemicondyles separated by a shallow trochlea (Fig. 9F). The medial hemicondyle is missing approximately its dorsal half, making its size relative to the lateral hemicondyle impossible to judge. The lateral hemicondyle extends 0.3 cm farther distally than the medial hemicondyle (Figs. 9A–9D), as in *Tyrannosaurus rex* (Brochu, 2003).

#### Supraacetabular crest of right ilium

**Figure 10:**
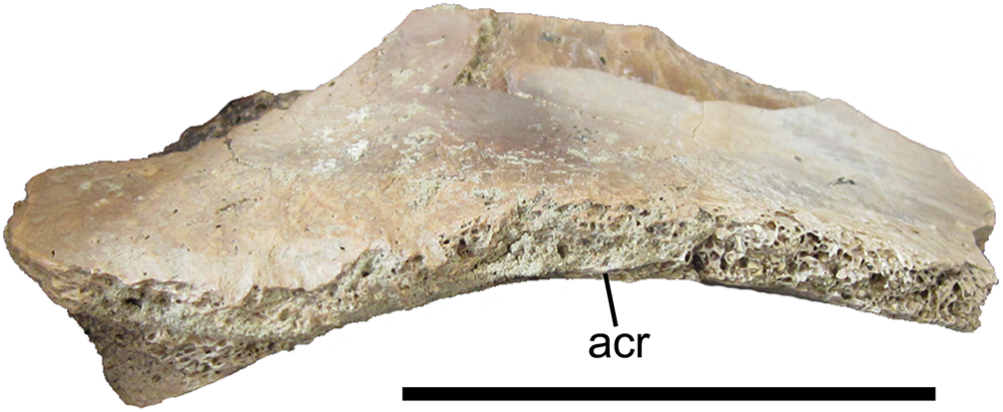
Supraacetabular crest of the right ilium of UMNH VP 28348. Supraacetabular crest of the right ilium in lateral view. Abbreviations: acr, rim of the acetabulum. Scale bar equals five cm.

A broadly arched bone fragment was identified as the supraacetabular crest of the right ilium, based upon comparisons with ilia of *Teratophoneus curriei* (UMNH VP 16690) and *Tyrannosaurus rex* (Fig. 92 in Brochu (2003)). The ventral surface of this fragment is smooth and gently concave to form the dorsal margin of the acetabulum. The lateral margin of the supraacetabular crest is very thin for most of its preserved length (0.7 cm thick at the midpoint of the preserved portion). However, the lateral margin becomes considerably thicker caudally (2.1 cm thick at its maximum preserved thickness), toward the base of the ischial peduncle (Fig. 10), as on the other tyrannosaurid ilia referenced above. The supraacetabular crest also becomes markedly thicker medially (2.5 cm thick at its maximum preserved thickness), as its dorsal margin slopes steeply dorsally to merge with the lateral surface of the blade of the ilium.

#### Phalanx 2 of left pedal digit IV

**Figure 11:**
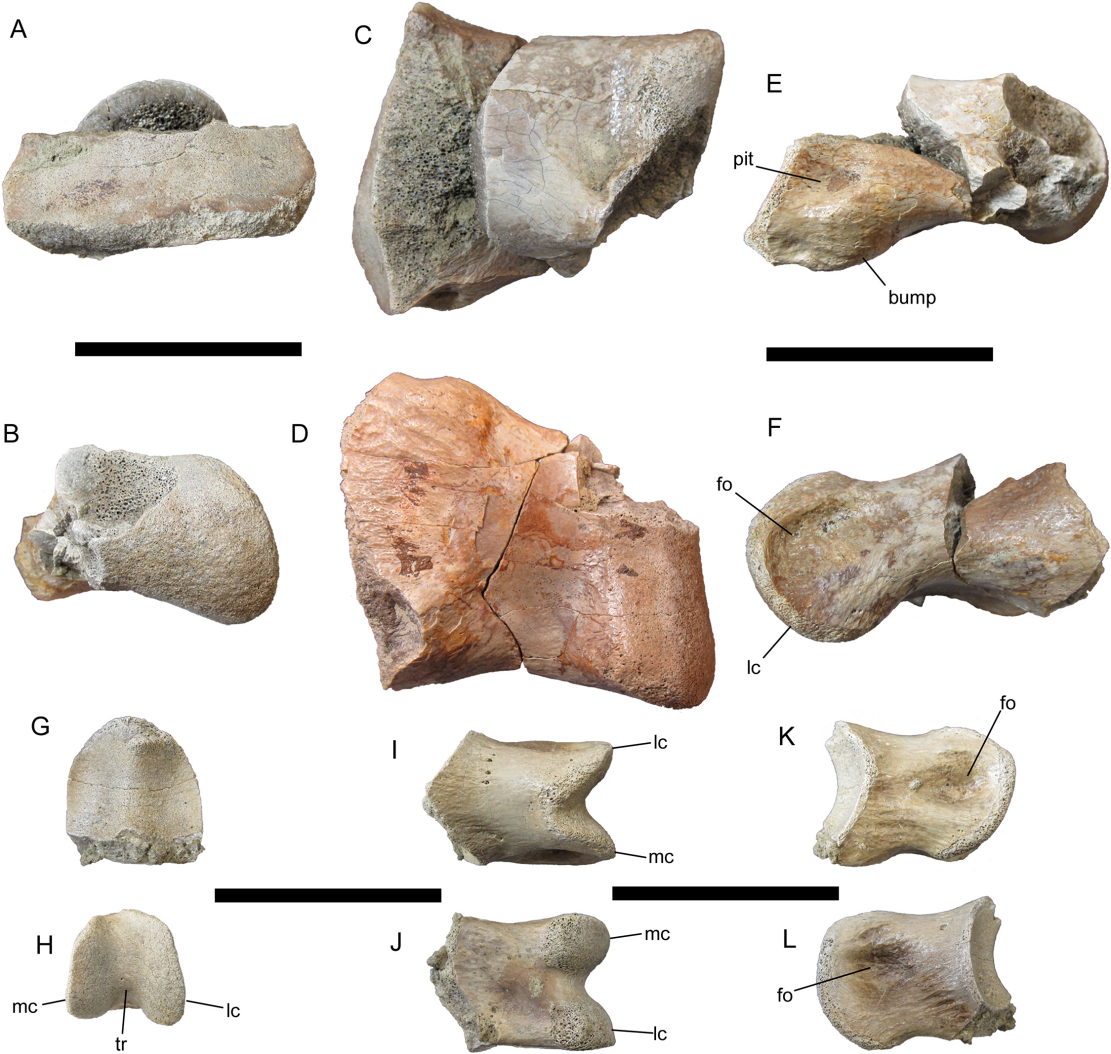
Pedal phalanges of UMNH VP 28348. Phalanx 2 of left pedal digit IV in (A) proximal; (B) distal; (C) dorsal; (D) ventral; (E) medial; and (F) lateral views. Phalanx 4 of left pedal digit IV in (G) proximal; (H) distal; (I) dorsal; (J) ventral; (K) medial; and (L) lateral views. Abbreviations: fo, fovea; lc, lateral condyle; mc, medial condyle; tr, trochlea. Scale bars equal five cm.

Two well-preserved phalanges were identified as phalanges 2 and 4 of left pedal digit IV based upon comparisons with LACM 23844, an articulated left pes of *Tyrannosaurus rex*, and the *Tyrannosaurus rex* pes described by Brochu (2003). IV-2 is missing the dorsal half of the proximal articular surface and the medial condyle (Figs. 11A–11F). The preserved portion of the proximal articular surface is subrectangular and divided into two facets by a broad, subtle ridge; the lateral facet is mediolaterally wider than the medial facet (Figs. 11A and 11C). Distal to the proximal articular surface, the shaft of IV-2 is constricted mediolaterally and dorsoventrally. On the medial surface of the shaft, immediately distal to the proximal articular surface, there is a deep circular pit with a pronounced bump ventral to it (Fig. 11E). The corresponding region on the lateral surface of the shaft is smooth and featureless (Fig. 11F). The lateral condyle is hemispherical and bears a deep fovea on its lateral surface (Fig. 11F).

#### Phalanx 4 of left pedal digit IV

IV-4 is much smaller than IV-2 in all dimensions (Figs. 11G–11L). As on IV-2, the proximal articular surface of IV-4 is divided into two facets; however, unlike those of IV-2, the facets of IV-4 are equal in size and demarcated by a distinct ridge (Fig. 11G). On the distal articular surface of IV-4, the medial and lateral condyles are separated by a much deeper trochlea than on IV-2 (Figs. 11H–11J). The lateral condyle is dorsoventrally deeper than the medial condyle. The medial and lateral condyles each bear a deep circular fovea, though the fovea on the lateral condyle is deeper than that on the medial (Figs. 11K and 11L), as in IV-4 of LACM 23844.

## Discussion

### Tyrannosaurid phylogeny

**Figure 12:**
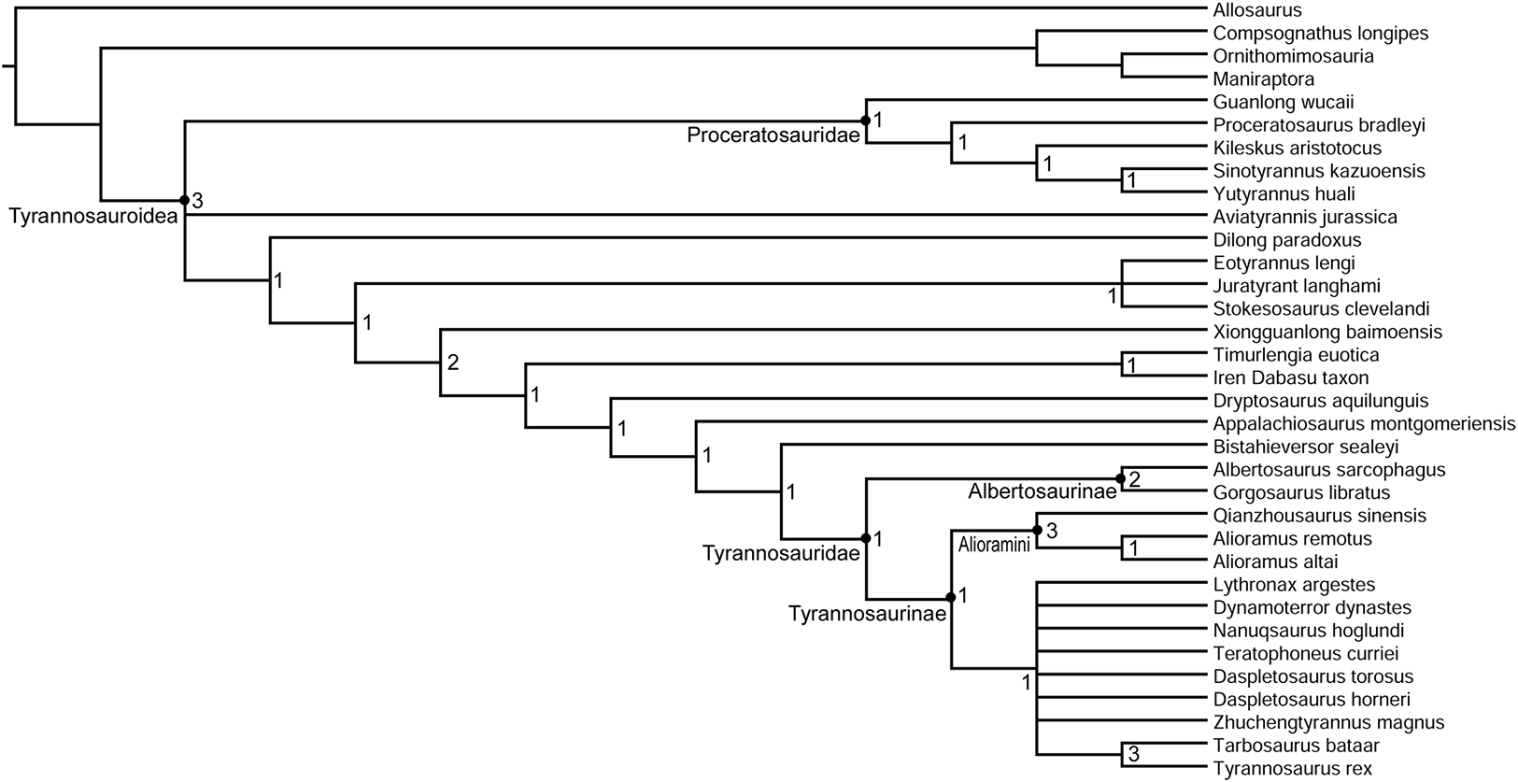
Phylogenetic relationships of *Dynamoterror dynastes*. Strict consensus cladogram of 18 most parsimonious trees obtained by TNT. Tree image was prepared in Mesquite. Bremer support values are next to each node.

The topology of the strict consensus cladogram (Fig. 12) is identical to that of Carr et al. (2017), except for a lack of resolution among large-bodied derived tyrannosaurines, including *Dynamoterror*, *Teratophoneus*, *Lythronax*, *Nanuqsaurus*, *Daspletosaurus*, *Zhuchengtyrannus*, and a clade composed of *Tarbosaurus* and *Tyrannosaurus*. This is probably due to the inclusion of the fragmentary new taxon *Dynamoterror*. Determining an area of origin for this large-bodied tyrannosaurine clade is difficult due to the paucity of the tyrannosauroid record from the Campanian of Asia (Brusatte & Carr, 2016) and the lack of diagnostic early tyrannosaurids from northern Laramidia. Two tyrannosaurids are now known from the lower Campanian of southern Laramidia, *L. argestes* from the Wahweap Formation of Utah (Loewen et al., 2013) and *Dynamoterror dynastes* from the Allison Member of the Menefee Formation of New Mexico. However, diagnostic tyrannosaurid material is currently lacking from roughly contemporaneous units in northern Laramidia, such as the lower Two Medicine Formation and McClelland Ferry Member of the Judith River Formation of Montana, and the Deadhorse Coulee Member of the Milk River Formation, Foremost Formation, and lower Oldman Formation of Alberta (Fowler, 2017). Furthermore, the early evolution and biogeography of Tyrannosaurinae in southern Laramidia remain enigmatic pending the discovery of additional tyrannosaurine material from the Menefee, Wahweap, and Kaiparowits formations. The current paucity of tyrannosauroid material from Santonian and lower Campanian units in Laramidia also hampers comparison with the tyrannosauroid record from Appalachia (Schwimmer et al., 1993, 2015; Carr, Williamson & Schwimmer, 2005; Brusatte, Benson & Norell, 2011; Brownstein, 2017; Dalman, Jasinski & Lucas, 2017).

### Reconstructing *Dynamoterror dynastes*

The dimensions of the frontals of UMNH VP 28348 are similar to those of LACM 23845, a subadult specimen of *Tyrannosaurus rex* (Currie, 2003; Carr & Williamson, 2004), suggesting roughly similar skull and body size, with the caveat that the proportions of these two individuals might have been different depending upon their relative ontogenetic stages. According to Molnar (1980), LACM 23845 is approximately 80% the size of an adult *Tyrannosaurus rex*. If UMNH VP 28348 was of similar size to LACM 23845, then this subadult or adult individual of *Dynamoterror dynastes* was about 30 feet long, a medium-sized tyrannosaurid.

**Figure 13:**
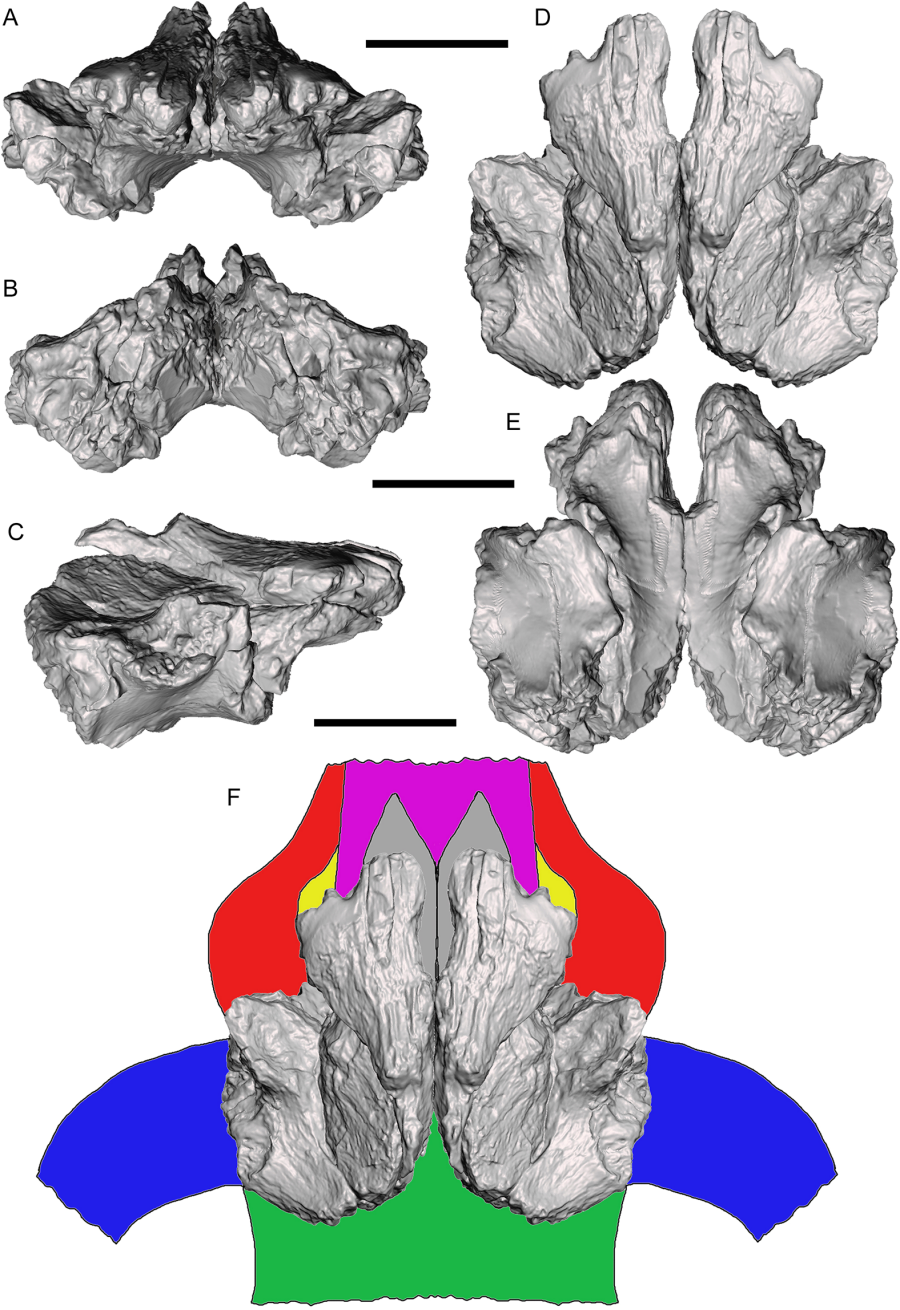
Reconstructed frontal complex of *Dynamoterror dynastes*. Composite, articulated right and left frontals in (A) rostral; (B) caudal; (C) right lateral; (D) dorsal; and (E) ventral views. (F) Reconstructed skull roof in dorsal view. Elements are color-coded as follows: frontals (gray); fused nasals (violet); prefrontals (yellow); lacrimals (red); postorbitals (blue); and parietal (green). Missing elements reconstructed based upon *Teratophoneus curriei* (UMNH VP 16690) (Loewen et al., 2013). Scale bars equal five cm.

Three-dimensional digital models of the described elements of UMNH VP 28348 were created by laser-scanning the bones. These models are available at MorphoSource under the project name “A new tyrannosaurid from the Upper Cretaceous Menefee Formation of New Mexico.” The models of the right and left frontals were aligned and combined on the basis of overlapping features, creating a composite pair that is nearly complete except for the nasal processes (Figs. 13A–13E). This allowed the frontal region of the skull roof to be reconstructed with some confidence based upon the positions, sizes, and morphologies of the nasal, prefrontal, lacrimal, postorbital, and parietal contacts (Fig. 13F). Several salient tyrannosaurine features can be observed on the reconstructed skull roof of *Dynamoterror*, such as large supratemporal fossae and a tall sagittal crest on the frontals, providing an expanded attachment area for enormous jaw-closing muscles (Currie, 2003; Carr & Williamson, 2004; Holliday, 2009).

## Conclusions

The description of *Dynamoterror dynastes* from the lower Campanian Allison Member of the Menefee Formation provides additional data on the morphology and diversity of early tyrannosaurines in Laramidia. However, additional discoveries are needed to elucidate the paleobiogeographic history of tyrannosaurines.

## Supplemental Information

10.7717/peerj.5749/supp-1 Supplemental Information 1 S1. Codings and measurements of UMNH VP 28348. Phylogenetic character codings and measurements of select cranial and appendicular elements of UMNH VP 28348.

## Institutional Abbreviations

BYU: Brigham Young University Museum of Paleontology, Provo, Utah, USA
LACM: Natural History Museum of Los Angeles County, Los Angeles, California, USA
UMNH: Natural History Museum of Utah (formerly Utah Museum of Natural History), Salt Lake City, Utah, USA.

## Additional Information and Declarations

### Field Study Permissions

The following information was supplied relating to field study approvals (i.e., approving body and any reference numbers): The specimen described herein was collected under permit NM12-03S issued by the U.S. Bureau of Land Management.

### Data Availability

The following information was supplied regarding data availability: The supplemental file includes a list of phylogenetic characters coded for the new taxon and a table of measurements of the fossil specimen.

### New Species Registration

The following information was supplied regarding the registration of a newly described species: Publication LSID: urn:lsid:zoobank.org:pub:E6547114-4285-49BB-AF82-1280F631748F Dynamoterror LSID: urn:lsid:zoobank.org:act:4B3FA975-DC43-4C1F-813F-29BB9DFDF9B6, *Dynamoterror dynastes* LSID: urn:lsid:zoobank.org:act:EED48535-DC9C-4E4A-A5BB-1ACA1D5FD918.
