# Supplementary material for: A new tyrannosaurid (Dinosauria: Theropoda) from the Upper Cretaceous Menefee Formation of New Mexico
Source: PeerJ. 2018 Oct 9;6:e5749. doi: 10.7717/peerj.5749 (PMC6183510; doi:10.7717/peerj.5749)
Supplement: Supplemental Information 1 — Phylogenetic character codings and measurements of select cranial and appendicular elements of UMNH VP 28348. [file peerj-06-5749-s001.docx]

**Codings for UMNH VP 28348.** *Dynamoterror dynastes* gen. et sp. nov. was coded for nine characters in the data matrix of Carr et al. (2017), as follows: 1^1^, 149^1^, 151^0^, 153^0^, 156^1^, 157^1^, 159^1^, 162^1^, and 385^1^.

**Measurements of UMNH VP 28348.** Measurements of select cranial and appendicular elements of UMNH VP 28348, holotype of *Dynamoterror dynastes* gen. et sp. nov. All measurements are in centimeters.

| **Elements** | **Measurements** |
| --- | --- |
|  |  |
| **Frontals** |  |
| Maximum width when frontals are joined along the midline interfrontal contact, measured between lateral-most points on the right and left postorbital sutures | 13.2 |
|  |  |
| **Right frontal** |  |
| Maximum preserved length, measured from the caudal-most point on the parietal suture to the rostral-most preserved point on the nasal process | 9.9 |
| Mediolateral width, from midline to medial edge of slot or groove between lacrimal and postorbital sutures (after Currie 2003) | 5.4 |
| Dorsoventral thickness, measured on midline at front of supratemporal fossa (after Currie 2003) | 3.1 |
| Maximum dorsoventral thickness at midline, measured from the dorsal margin of the sagittal crest to the ethmoid scar | 4.8 |
| Rostrocaudal length of the base of the sagittal crest | 5.5 |
| Maximum mediolateral width of prefrontal suture | 1.5 |
| Total preserved rostrocaudal length of postorbital suture | 4.4 |
| Rostrocaudal length of rostral portion of postorbital suture | 3.2 |
| Rostrocaudal length of caudal portion of postorbital suture | 1.3 |
| Rostrocaudal length of orbital wall, measured from caudal-most point on the lacrimal suture to the laterosphenoid suture | 4.5 |
| Dorsoventral depth of orbital wall, measured from ventral-most point on the postorbital suture to the ventral-most point on the crista cranii | 4.7 |
| Rostrocaudal length of the olfactory bulb fossa, measured from the ethmoid scar to the fossa’s caudal-most point | 4.2 |
| Mediolateral width of the olfactory bulb fossa, measured from the midline to the orbitosphenoid suture | 2.1 |
|  |  |
| **Left frontal** |  |
| Mediolateral width, from midline to medial edge of slot or groove between lacrimal and postorbital sutures (after Currie 2003) | 6.2 |
| Total preserved rostrocaudal length of postorbital suture | 5.1 |
| Rostrocaudal length of rostral portion of postorbital suture | 3.5 |
| Rostrocaudal length of caudal portion of postorbital suture | 1.1 |
|  |  |
| **Right metacarpal II** |  |
| Maximum proximodistal length, from proximal articular surface to lateral hemicondyle (after Brochu 2003) | 5.1 |
| Maximum mediolateral width of proximal articular surface | 1.4 |
| Maximum dorsoventral depth of proximal articular surface | 2.5 |
| Maximum mediolateral width of distal articular surface | 1.8 |
| Maximum dorsoventral depth of lateral hemicondyle | 1.2 |
|  |  |
| **Phalanx 2 of left pedal digit IV** |  |
| Maximum proximodistal length, from proximal articular surface to lateral condyle | 6.4 |
| Mid-shaft width (after Brochu 2003) | 5.0 |
| Maximum mediolateral width of proximal articular surface | 6.5 |
| Maximum dorsoventral depth of lateral condyle | 3.3 |
|  |  |
| **Phalanx 4 of left pedal digit IV** |  |
| Maximum proximodistal length, from proximal articular surface to lateral condyle | 3.0 |
| Mid-shaft width (after Brochu 2003) | 2.7 |
| Maximum mediolateral width of proximal articular surface | 2.8 |
| Maximum dorsoventral depth of proximal articular surface | 2.9 |
| Maximum mediolateral width of distal articular surface | 2.8 |
| Maximum dorsoventral depth of lateral condyle | 2.9 |
| Maximum dorsoventral depth of medial condyle | 2.6 |
